# Crystal structure of the feruloyl esterase from *Lentilactobacillus buchneri* reveals a novel homodimeric state

**DOI:** 10.3389/fmicb.2022.1050160

**Published:** 2022-12-08

**Authors:** Kamyar Mogodiniyai Kasmaei, Dayanand C. Kalyani, Tom Reichenbach, Amparo Jiménez-Quero, Francisco Vilaplana, Christina Divne

**Affiliations:** ^1^Department of Industrial Biotechnology, School of Engineering Sciences in Chemistry, Biotechnology, and Health (CBH), KTH Royal Institute of Technology, Stockholm, Sweden; ^2^Department of Animal Nutrition and Management, Swedish University of Agricultural Sciences, Uppsala, Sweden; ^3^Division of Glycoscience, Department of Chemistry, School of Engineering Sciences in Chemistry, Biotechnology, and Health (CBH), KTH Royal Institute of Technology, Stockholm, Sweden

**Keywords:** feruloyl esterase, ferulic acid, *Lentilactobacillus buchneri*, crystal structure, arabinoxylan

## Abstract

Ferulic acid is a common constituent of the plant cell-wall matrix where it decorates and can crosslink mainly arabinoxylans to provide structural reinforcement. Microbial feruloyl esterases (FAEs) specialize in catalyzing hydrolysis of the ester bonds between phenolic acids and sugar residues in plant cell-wall polysaccharides such as arabinoxylan to release cinnamoyl compounds. Feruloyl esterases from lactic acid bacteria (LAB) have been highlighted as interesting enzymes for their potential applications in the food and pharmaceutical industries; however, there are few studies on the activity and structure of FAEs of LAB origin. Here, we report the crystal structure and biochemical characterization of a feruloyl esterase (*Lb*FAE) from *Lentilactobacillus buchneri*, a LAB strain that has been used as a silage additive. The *Lb*FAE structure was determined in the absence and presence of product (FA) and reveals a new type of homodimer association not previously observed for fungal or bacterial FAEs. The two subunits associate to restrict access to the active site such that only single FA chains attached to arabinoxylan can be accommodated, an arrangement that excludes access to FA cross-links between arabinoxylan chains. This narrow specificity is further corroborated by the observation that no FA dimers are produced, only FA, when feruloylated arabinoxylan is used as substrate. Docking of arabinofuranosyl-ferulate in the *Lb*FAE structure highlights the restricted active site and lends further support to our hypothesis that *Lb*FAE is specific for single FA side chains in arabinoxylan.

## Introduction

Ferulic acid (FA; 4-hydroxy-3-methoxy-cinnamic acid) is widely distributed in nature as a structural reinforcement component of plant cell walls (Lambert et al., 1999), where it is incorporated into the cell wall matrix by esterifying hemicelluloses, mainly glucuronoarabinoxylans (Carpita, 2001), as well as forming ether bonds to lignin and proteins (Kondo et al., 1990). Additionally, ferulic acid can form cross-links between lignin and arabinoxylan (AX) chains to produce lignin-ferulate-polysaccharide complexes (Terrett and Dupree, 2019). Furthermore, diferulate dimers can form cross-links between individual AX chains (Ralph et al., 1994). Agricultural residues constitute an important source of renewable carbon, containing some 0.5 to 3.0% FA (w/w), and these residues are therefore promising sources for FA production (Zhao and Moghadasian, 2008). Several methods have been developed to produce FA, including chemical and enzymatic hydrolysis (Antonopoulou et al., 2022). Nevertheless, enzymatic extraction catalyzed by feruloyl esterase (FAE) is more efficient, safe, and environmentally friendly than chemical extraction methods that commonly use harsh alkali solvents (Liu et al., 2021).

Feruloyl esterases (FAEs; E.C. 3.1.1.73) or cinnamoyl esterases (CEs), have been classified in the CAZy database as belonging to the carbohydrate esterase (CE) family (Drula et al., 2022). They are hydrolases belonging to the subclasses of carboxylic-acid esterases that catalyze the hydrolysis of ester bonds between phenolic acids (ferulic acid, *p*-coumaric acid, etc.) and sugar residues in plant cell-wall polysaccharides (arabinoxylan, pectin, etc.). FAEs are attractive due to their broad range of potential applications in biofuel, food, pharmaceutical and paper and pulp industries (Koseki et al., 2009; Dilokpimol et al., 2016). In the presence of FAE, feruloylated AX fibers are de-esterified into FA, di-FA, and the AX polysaccharide core, which have antioxidant, anti-inflammatory, and antimicrobial properties (Oliveira et al., 2019). The configuration of plant cell walls can be disrupted by FAEs acting on the lignin-ferulate-polysaccharide complexes, and thereby enhancing bioavailability of structural carbohydrates (cellulose and hemicelluloses) for enzymatic hydrolysis, e.g., in the rumen, biogas reactors, etc. (Tabka et al., 2006). Microbial FAEs were originally classified into four subfamilies, A to D, mainly based on substrate preference and the ability to release 5,5’-diFA from plant cell walls (Crepin et al., 2004). More recently, several groups have further expanded the classification (Benoit et al., 2008; Dilokpimol et al., 2016, 2017, 2018; Udatha et al., 2011, 2012). Of these reports, only Udatha et al. (2011) have offered some information regarding the classification of FAEs of bacterial and plant origins.

FAEs of fungal origin are currently the best characterized, and of considerable industrial interest (Dilokpimol et al., 2016; Underlin et al., 2020). However, several bacterial FAEs have also been characterized structurally and biochemically, including the FAEs from *Clostridium thermocellum* (Tabouriech et al., 2005), *Dickeya dadantii* (Hassan and Hugouvieux-Cotte-Pattat, 2011), Lactobacilli and Bifidobacteria (Lai et al., 2011; Fritsch et al., 2017; Xu et al., 2017; Zhang et al., 2021), *Butyrivibrio proteoclasticus* (Goldstone et al., 2010), *Bacteroides intenstinalis* (Pereira et al., 2021), *Streptomyces cinnamoneus* (Uraji et al., 2018), and uncultured bacteria from metagenomic studies (Holck et al., 2019).

FAEs derived from Lactobacillus or lactic acid bacteria (LAB) constitute an intriguing group of enzymes with potential applications in the food and pharmaceutical industries (Xu et al., 2020). In addition to having beneficial effects on the human and animal intestinal tracts (Wang et al., 2004; Liu et al., 2021), these bacteria also play an important role in silage fermentation, which relies on the production of organic acids to inhibit the growth of unwanted microorganisms (Mogodiniyai Kasmaei et al., 2020). Several LABs demonstrate high FAE activity, including *L. acidophilus*, *L. amylovorus*, *L. farciminis*, *L. fermentum*, *L. gasseri*, *L. helveticus*, *L. johnsonii*, and *L. reuteri*, *L. farciminis*, *L. reuteri*, and *L. crispatus* (Wang et al., 2004; Lai et al., 2011; Esteban-Torres et al., 2013; Xu et al., 2020; Liu et al., 2021; Zhang et al., 2021), but few studies have investigated the structural basis of the substrate binding and catalytic mechanisms.

In the present study, we expressed and purified FAE from *Lentilactobacillus buchneri* LN 4017 (*Lb*FAE) and characterized its enzymatic properties. The crystal structure of *Lb*FAE was determined in complex with FA, which allowed identification of the key residues responsible for FA binding, as well as predicting the binding mode of arabinofuranosyl ferulate (Araf-FA) binding. The present work offers new insight regarding the structural determinants of the substrate specificity and activity for *Lb*FAE and provides a basis for further protein engineering for biotechnological applications.

## Materials and methods

### Substrates

Methyl ferulate (MF), methyl sinapinate and *p*-nitrophenyl ferulate (*p*NPF) were procured from Carbosynth Ltd. (United Kingdom). Methyl caffeate and methyl trans-*p*-coumarate were purchased from Tokyo Chemical Industry (USA). Polymeric feruloylated arabinoxylan (F-AX) was extracted from corn bran as described below and used as natural substrate.

### Cloning and site-directed mutagenesis

The gene coding for a FAE annotated as a type-A FAE (*faeA*) from *L. buchneri* silage additive strain LN4017 (ATCC PTA-6138; GenBank ADI72729; UNP D7RU28) was synthesized by Integrated DNA Technologies (IDT, Iowa, USA), and cloned into the vector pNIC28-Bsa4 using ligation-independent cloning (LIC) technique. The *Lb*FAE gene was amplified using the following forward and reverse primers:

Forward primer: 5′- TACTTCCAATCC ATGATCAAATTTGTAACTAC-3′.

Reverse primer: 5′- TATCCACCTTTACTG TTATTTTGCTTCTAACAACG-3′.

The 50 μl PCR mixture contained 50 ng plasmid DNA, 2 U Phusion High-Fidelity DNA polymerase (Thermo Fisher), 0.5 μM of each primer, 200 μM dNTP, 1.5 μl DMSO and 1 x HF Phusion buffer (Thermo Fisher). Thermocycling program was an initial denaturation at 98°C for 30 s followed by 30 cycles of denaturation (98°C for 10 s), annealing (60°C for 30 s) and elongation (72°C for 90 s) before a final elongation at 72°C for 10 min. PCR products were treated with 10 U of DpnI (Thermo Fisher) to digest methylated copies of DNA before purification with GeneJET PCR Purification Kit (Thermo Fisher). After linearization of pNIC28-Bsa4 vector with Eco31I (Thermo Fisher) and treatment of vector and insert with T4 DNA Polymerase to create overhang, the insert was annealed into the vector. This was followed by transforming the vector into chemically competent *E. coli* DH5α cells (Invitrogen) and plating on Luria-Bertani (LB) agar fortified with 50 μg ml^−1^ kanamycin at 37°C for 16 h.

The catalytic serine (Ser114) was replaced by alanine using site-directed mutagenesis to generate the *Lb*FAE variant S114A. Site-directed mutagenesis was performed with the QuikChange II site-directed mutagenesis kit (Agilent Technologies) as described by the manufacturer, and using the following forward and reverse primers:

S114A _fwd (5′- CATTGCTGGGCGAA*GCA*CTGGGTAGTGTTGC-3′).

S114A _rev (5′- GCAACACTACCCAG*TGC*TTCGCCCAGCAATG-3′).

The wild-type gene sequence was used as template for generating the S114A mutant. The mutated gene sequence was confirmed by DNA sequencing before transformation into *E. coli* BL21 (DE3)-T1 for protein production.

### Production and purification of *Lb*FAE

The recombinant plasmids (wild type and S114A variant) were transformed into *E. coli* BL21(DE3)-T1 competent cells and the bacteria was cultured in Terrific Broth supplemented with 50 μg ml^−1^ kanamycin at 37°C. After reaching an OD_600_ value of ~0.6, the temperature was reduced to 17°C and when the OD_600_ value reached ~1.0, *β*-D-1-thiogalactopyranoside (IPTG) was added at 0.2 mM to induce FAE gene expression. After ~18 h incubation, bacterial cells were harvested by centrifugation at 4500 × *g* (Beckman Coulter JA-10 fixed-angle rotor). The bacterial cells were resuspended in a buffer containing 25 mM K_2_HPO_4_ (pH 7.2), 150 mM NaCl and 5% (v/v) glycerol with one tablet of cOmplete™ Protease Inhibitor Cocktail (Roche) before homogenization with AVESTIN Emulsiflex-C3 (Avestin Europe, GmbH), and centrifugation (Beckman Coulter Avanti J-20 XP, California, USA) at 12,096 × *g* for 10 min at 4°C.

The Ni-NTA agarose resin was equilibrated with buffer containing 25 mM K_2_HPO_4_ (pH 7.2), 150 mM NaCl and 5% (v/v) glycerol. The supernatant was incubated with equilibrated Ni-NTA agarose resin for 1 h at 4°C and then loaded onto Bio-Rad Econo-Pac. The bound proteins to the resin was first washed with a buffer containing 25 mM K_2_HPO_4_ (pH 7.2), 150 mM NaCl, 30 mM imidazole and 5% (v/v) glycerol and thereafter with a buffer containing 25 mM K2HPO4 (pH 7.2), 300 mM NaCl, 50 mM imidazole and 5% (v/v) glycerol before eluting bound proteins with an elution buffer (25 mM K_2_HPO_4_ pH 7.2) 150 mM NaCl, 500 mM imidazole and 5% (v/v) glycerol). Washing and elution were carried out at 4°C.

The eluted proteins were concentrated using Vivaspin20 centrifugal concentrators (polyethersulfone filter, 10 kDa molecular weight cut-off) and were subjected to size exclusion chromatography using HiLoad 16/60 Superdex 200 prep grade column (GE Healthcare Life Sciences) and a mobile phase containing 50 mM 4-(2-hydroxyethyl)-1-piperazineethanesulfonic acid (Hepes) pH 7.5, 150 mM NaCl, and 5% (v/v) glycerol. The recovered fractions of FAE were analyzed using SDS-PAGE and pooled, and further concentrated with a Vivaspin20 centrifugal spin concentrator. Approximately 27 mg of recombinant *Lb*FAE was obtained from 1 liter of bacterial culture.

### Optimal pH, optimal temperature, substrate specificity and kinetic parameters

The optimal pH and temperature of *Lb*FAE activity were determined using MF as substrate. A 200 μl mixture contained 102 μg ml^−1^ *Lb*FAE and 100 μM MF and 1% DMSO. The optimal pH was evaluated in a buffer system comprising three buffers: 100 mM citrate–phosphate (pH 4.0, 4.5, 5.0, 5.5, 6.0); 100 mM sodium phosphate (pH 6.0, 6.5, 7.0, 7.5, 8.0); and 100 mM Tris–HCl (pH 8.0, 8.5, 9.0).

The reaction was carried out at 30°C for 10 min and terminated by placing the reaction in a boiling water bath for 5 min. Released FA was quantified using HPLC and a Photodiode Array Detector at 319 nm (Waters Assoc., USA). The column used was Luna Omega PS C18 (250 × 4.6 mm, particle size 5 μm; Phenomenex Inc., Torrance, USA). The column temperature was set at 30°C. Acetonitrile:acetic acid (10%) 20:80 (v:v) was the mobile phase. The optimal temperature of *Lb*FAE was determined in sodium-phosphate buffer (100 mM, pH 6.5) for the temperature range 10–70°C with samples taken at 10°C intervals and analyzed as above.

The catalytic activities of *Lb*FAE against methyl p-coumarate, methyl caffeate and methyl sinapinate and S114A *Lb*FAE against MF were evaluated at the optimal pH and temperature determined for MF. The FAE activity using *p*NPF substrate was measured spectrophotometrically as described previously (Mastihuba et al., 2002). The kinetic constants (*V*_max_, *K*_m_) for MF and *p*NPF were calculated using a 200 μl^−1^ reaction mixture including 10 or 50 μg ml^−1^ *Lb*FAE and 0.0125–2.0 mM of substrate at the derived optimal pH and temperature. *V*_max_ and *K*_m_ were determined by fitting the Michaelis–Menten equation to the data using nonlinear regression (*R*^2^ = 0.9724) in GraphPad Prism 5 program (GraphPad Software, San Diego California USA). All assays were performed in triplicates.

### Degradation of feruloylated arabinoxylan

Feruloylated arabinoxylan (F-AX) was extracted from corn bran (provided by Cargill Deutschland GmbH, Krefeld, Germany from their wet steeping process of corn grain) and characterized as reported previously (Rudjito et al., 2020). The de-starching step and the subcritical water extraction (SWE) of F-AX were performed at the pilot plant by Celabor (Chaineux, Belgium). Phenolic acid profile of F-AX was determined after saponification and HPLC-UV as described previously (Rudjito et al., 2020).

The F-AX was solubilized in phosphate buffer (100 mM, pH 6.5) at 5 mg ml^−1^. The enzyme was diluted in phosphate buffer and added into 2 ml substrate medium (in triplicate) to reach a concentration of 1 mg enzyme per g of F-AX. The reaction was carried out at 40°C and aliquots (100 ul) were taken after 10 min, 2, 4, 8, and 24 h, directly inactivated at 100°C for 5 min and stored in dark at 4°C for the analysis of phenolic compounds.

Sinapic acid, *p*-coumaric acid, ferulic acid, and diferulic acids (5–5′ di-FA and 8–8′ di-FA) were quantified by HPLC as described previously (Rudjito et al., 2020). In short, aliquots were diluted 5-times in methanol:2% acetic acid mixture (1.1 v/v) and 10 μl were injected (1 ml min^−1^) onto the HPLC system (Waters 2,695 separation module, USA) equipped with a C18 guard column and an SB-C18 separation column (Zorbax SB-C18 5 μm particle size, 4.6 × 250 mm, Agilent, USA). The separation was performed at 25°C using as eluent A 2% acetic acid in H2O (v/v); and eluent B 100% methanol. The gradient used for the separation was: 100%–75% A (11 min), 71.25% A (4 min), 64% A (10 min), 55% A (10 min), 35% A (3 min), 100% A (3 min) and 100% A (4 min), and measurements were made on a photodiode array detector (Waters 2,996, USA) at 200–400 nm. Concentrations between 0.005 g l^−1^ and 0.1 g l^−1^ were used for standard calibration.

### Analysis of oligomeric state using size-exclusion chromatography

The oligomeric state of *Lb*FAE was investigated using size-exclusion chromatography (SEC) on a HiLoad 16/60 Superdex 200 prep grade column equilibrated with 20 mM Hepes (pH 7.5) containing 150 mM NaCl at a flow rate of 1 ml min^−1^ at 4°C. For estimation of the *Lb*FAE molecular weight, the standard proteins used were ferritin (440 kDa), aldolase (158 kDa), conalbumin (75 kDa), and ribonuclease A (13.7 kDa).

### Protein crystallization and structure determination

*Lb*FAE could be crystallized under several conditions using vapor diffusion in hanging drops at room temperature. Crystals of wild-type and S114A *Lb*FAE were obtained under two different reservoir conditions, either by mixing 1 μl 20 mg ml^−1^ protein in 50 mM Hepes pH 7.5, 150 mM NaCl, 5% (v/v) glycerol with 0.5 μl reservoir solution 1 (0.1 M 2,2-Bis(hydroxymethyl)-2,2′,2″-nitrilo-triethanol (Bis-Tris) buffer (pH 5.5), 0.2 M sodium chloride, 25% (w/v) polyethylene glycol (PEG) 3,350); or reservoir solution 2 (0.1 M sodium cacodylate (pH 6.5), 0.2 M calcium acetate and 18% (w/v) PEG 8000). The S114A crystals used for data collection were soaked with either methyl ferulate (MF) or methyl sinapinate (MS) prior to harvest and flash-freezing. All crystals were flash-frozen in liquid nitrogen prior to data collection.

X-ray diffraction data were collected using synchrotron radiation, and the *XDS* package was used for processing and scaling (Kabsch, 2010). As mentioned above, *Lb*FAE crystallizes in different space groups, but without a clear correlation to the crystallization conditions used. A tetragonal wild-type dataset (1.9 Å resolution) was phased by molecular replacement with *PHENIX Phaser* (Adams et al., 2010) using the prepared homology model. A clear solution was obtained, and the phases were further improved by density modification using *RESOLVE* implemented in the *PHENIX* suite. A complete initial model could be built, and further improved by iterative manual model correction using *COOT* (Emsley et al., 2010) guided by *σ*_A_-weighted 2*F*_o_-*F*_c_ electron-density maps and crystallographic refinement in *PHENIX*. All model refinement was performed using *phenix.refine* and included reciprocal-space refinement of *x*, *y*, *z* coordinates, and individual *B* factor refinement.

The refined, initial model was used to phase three datasets using molecular replacement with *PHENIX* (Supplementary Table S1): a 1.90-Å resolution dataset from a wild-type crystal (reservoir 2) soaked with a grain of FA added to the mother liquor prior to crystal harvest; a 1.50-Å dataset of the wild type without ligand (reservoir 1); and a 1.45-Å dataset of the S114A variant soaked with MS (reservoir 2). The resulting molecular-replacement solutions generated models that were manually corrected and refined as described above. Although several *Lb*FAE S114A crystals soaked with MF or MS were screened, no structure with bound MF or MS could be identified, and only the unliganded mutant structure was refined.

Electrostatic potential surfaces were calculated using *APBS-PDB2PQR* software suite at the PoaissonBolzmann server (Jurrus et al., 2018)^1^. *PDB2PQR* was run using *PROPKA* to assign protonation states at pH 6.5, and the PARSE forcefield. The output was used as input to *APBS* calculations using automatically configured finite difference Poisson-Boltzmann calculations (*mg-auto*). Electrostatic potential surfaces were visualized with *PyMOL* (The PyMOL Molecular Graphics System, Version 2.0 Schrödinger, LLC).

### Sequence analysis of FAEs with known 3D structure

Sequences for the putative FAEs listed in Supplementary Table S2 with known three-dimensional (3D) structure were structurally aligned using the multiple structure 3D alignment algorithm in *PDBeFold v.2.59* (Krissinel and Henrick, 2004)^2^, and the aligned multiple sequence alignment (MSA) used as input for generating a phylogenetic tree. The MSA was visualized using ESPript 3.0 (Robert and Gouet, 2014)^3^. A phylogenetic tree was computed using the *Simple Phylogeny* option at the EMBL-EBI server (Madeira et al., 2019)^4^ using the pre-aligned sequences as input. The tree was visualized using *iTOL v.6.4.3* (Interactive Tree of Life^5^; Letunic and Bork, 2021). An alignment was also generated using *PDBeFold v.2.59* including only the closest relatives (LJ0536 and Est1E), also visualized using ESPript 3.0.

## Results

### SEC analysis of the oligomeric state

The theoretical molecular weight of *Lb*FAE including the His6 tag and TEV-cleavage site is 31.6 kDa, which was confirmed as a single band of approximately 30 kDa by SDS-PAGE analysis (Supplementary Figure S1), and a SEC elution profile that is consistent with a molecular mass of approximately 60 kDa (Supplementary Figure S1), which provides support for a homodimeric state.

### Characterization of the catalytic activity of *Lb*FAE

**Figure 1:**
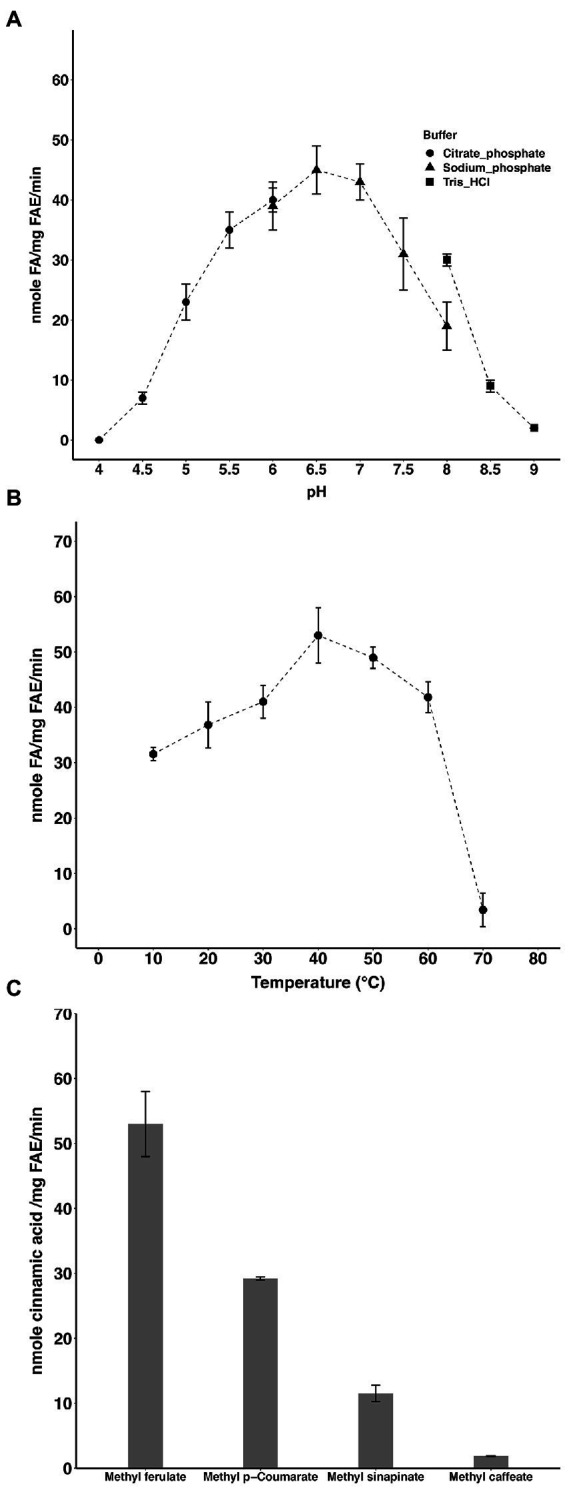
Activity of *Lb*FAE. pH **(A)** and temperature **(B)** optimum of activity with MF as substrate. **(C)** Activity on different hydroxycinnamic acids. Measurements were performed in triplicates and the error bars indicate the mean ± SD.

The optimal pH and temperature of the enzyme were 6.5 and 40°C, respectively (Figures 1A,B). *Lb*FAE released (per mg of enzyme) 53 nmol min^−1^ FA, 29 nmol min^−1^ *p*-coumaric acid, and 12 nmol sinapic acid min^−1^ from the corresponding methyl esters under the experimental conditions used (Figure 1C). The lowest activity was observed for methyl caffeate where only 2 nmol min^−1^ (per mg enzyme) was released. Further, the S114A *Lb*FAE showed no catalytic activity on MF.

**Figure 2:**
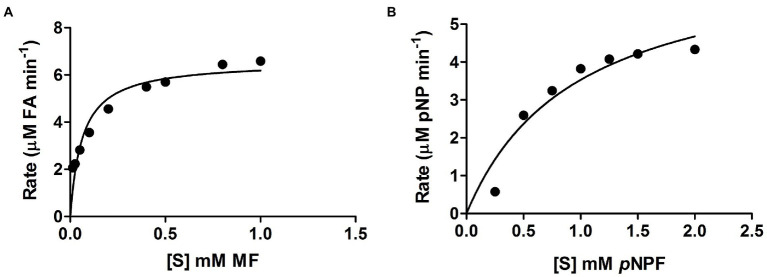
Velocity versus substrate concentration. **(A)** Methyl ferulate (MF), and **(B)** *p*-nitrophenyl ferulate (pNPF). Values are shown as the mean and SD for triplicate experiments.

Curves showing the enzyme velocity versus substrate concentration for *Lb*FAE toward the MF and *p*NPF (Figure 2) demonstrate a significant difference in velocity of the enzyme reaction with the two substrates. The kinetic parameters (*k*_cat_ and *K*_m_) were determined from the fit of the Michalis-Menten reaction to the data (Table 1). *Lb*FAE shows an apparent higher affinity for MF than *p*NPF, as the estimated *K*_m_ is 13-fold lower for MF than *p*NPF. The *k*_cat_/*K*_m_ ratio shows that *Lb*FAE hydrolyses MF 34 times more efficiently than *p*NPF. The high *k*_cat_/*K*_m_ ratio for MF is attributed to the very fast reaction (high *k*_cat_) and the high substrate affinity (low *K*_m_) as compared to *p*NPF.

**Table 1:** Kinetic parameters of *Lb*FAE determined against the *p*NPF and MF.

Substrate	*K_m_* (mM)	*V*_*max*_ (U mg^−1^)	*K_cat_* (s^−1^)	*K_cat_/K_m_* (s^−1^ mM)
*p*NPF	0.9	6.9	6.7	7.54
MF	0.065	6.57	16.3	252

### Degradation of feruloylated arabinoxylan

**Figure 3:**
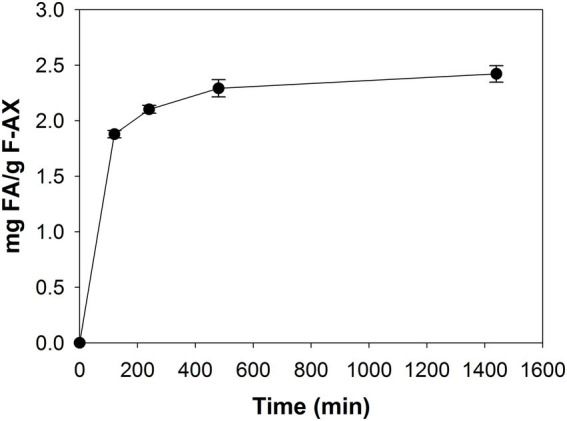
Degradation of feruloylated arabinoxylan. Release of FA from F-AX by incubation with *Lb*FAE for 24 h. Error bars indicate the mean ± SD.

The hydroxycinnamic-acid profile of F-AX after saponification was as follows: 26.6 mg FA; 1.4 mg *p*-coumaric acid; 0.6 mg sinapic acid; 1.1 mg 5–5′ diferulic acid; and 0.7 mg 8–8′ diferulic acid. Per g substrate. FA was the only phenolic component detected from F-AX after treatment with *Lb*FAE (Figure 3). We did not detect *p*-coumaric acid, sinapic acid or FA dimers. After 2 h incubation, *Lb*FAE released 1.9 mg FA per g substrate, increasing slightly to 2.4 mg after 24 h incubation, corresponding to 9% of the total amount of FA present in F-AX.

### Overall structure of *Lb*FAE

Wild-type crystals grew as either triclinic (*P*1) or tetragonal (*P*4_3_2_1_2) crystals under identical crystallization conditions, whereas crystals of the S114A variant were isomorphous with the *P*4_3_2_1_2 crystal form despite being produced under different conditions. Several *Lb*FAE crystals were screened, and one dataset of the wild type was used for initial phase determination. A homology model was produced manually based on aligning the *Lb*FAE sequence with that of the crystal structure of *Lactobacillus johnsonii* cinnamoyl esterase LJ0536 (PDB 3PFB; Lai et al., 2011) and mutating the amino acids of the template in *COOT* (Emsley et al., 2010).

**Figure 4:**
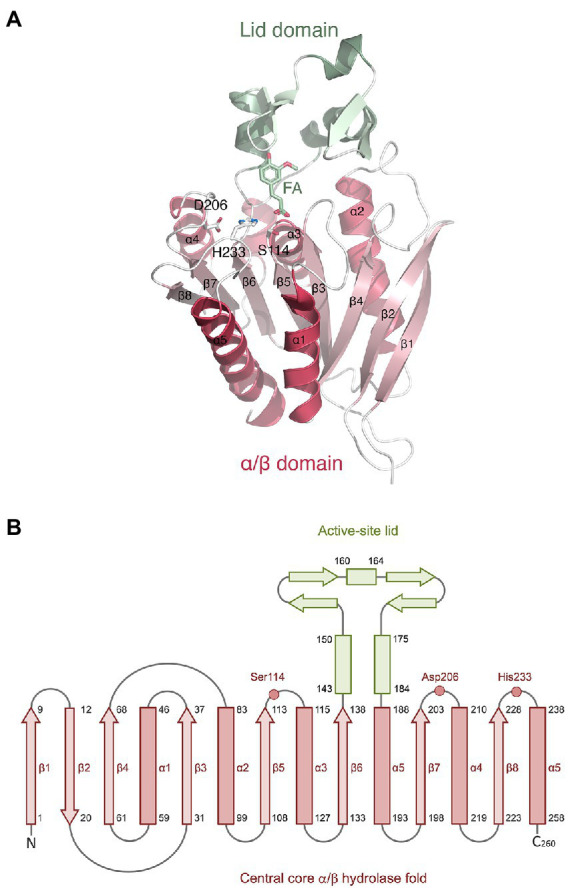
Monomer structure of *Lb*FAE. **(A)** Ribbon drawing showing the eight-stranded α/β hydrolase fold (red) and the lid domain (green). Ferulic acid (FA) and the side chains of the catalytic triad (Ser114, His233, Asp206) are shown as ball-and-stick objects. **(B)** Topology diagram showing the same information as in **(A)**.

The *Lb*FAE monomer structure includes 260 amino acids that forms the canonical α/β-hydrolase superfamily fold with a conserved Ser-His-Asp catalytic triad (Figure 4). The structure is characterized by a central open twisted β-sheet with eight β-strands starting with an N-terminal β-hairpin followed by six parallel β-strands, which includes three complete βαβ motifs. The active site is located at the C-terminal ends of the parallel β-strands of the α/β-hydrolase core where the crossover connection between βαβ motifs occur. An extensive domain (residues 140–186), inserted between β5 and α5, folds over the active site to form a large “lid.” The conserved catalytic triad (Figure 4) is situated at the end of β5 (Ser114), and in the loops following β8 (His233) and β7 (Asp206). The nucleophilic serine (Ser114 in *Lb*FAE) is positioned at the center of a conserved lipase (EC 3.1.1.3)-esterase (EC 3.1.1.1) pentapeptide G-X-S-X-G motif (Brenner, 1988).

**Figure 5:**
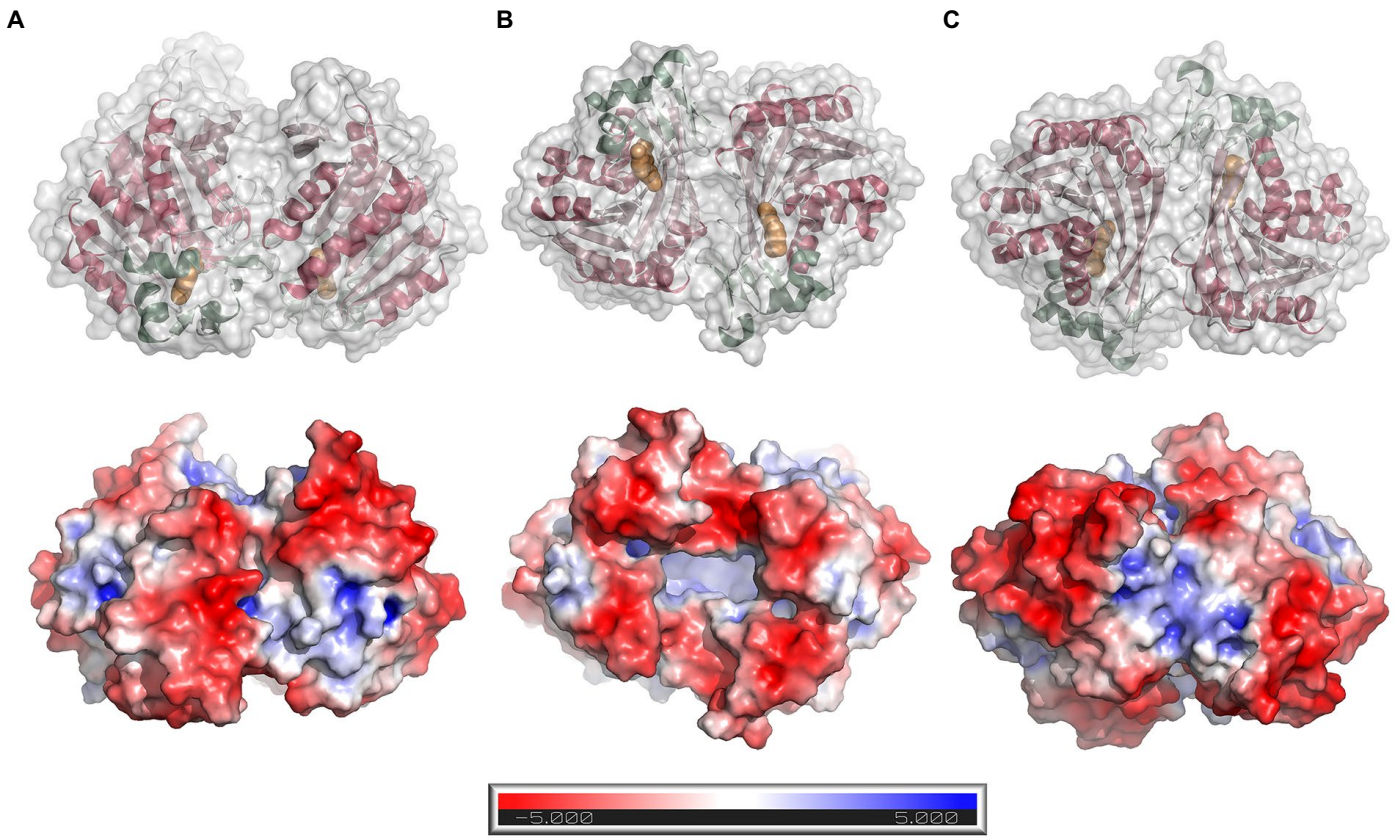
Dimer structure and surface electrostatics. Top row: ribbon drawings with an overlaid semitransparent gray molecular surface showing the *Lb*FAE homodimer in three orientations **(A)** side view, **(B)** bottom view, and **(C)** top view. The α/β domain is colored red, and the lid domain is green. Ferulic acid is shown in orange. Bottom row: electrostatic potential surfaces calculated at pH 6.5 and colored in the range − 5.0 to 5.0 kT/e. The entrance to the active site is located at the bottom of the dimer, which is emphasized in orientation B.

The protein forms a homodimer with two-fold symmetry where the active sites from the two monomers are oriented facing each other (Figure 5A). The active sites can be accessed only through a groove formed between the two monomers at one end of the homodimer (the opening to the groove is seen in the bottom view in Figure 5B). The homodimeric structure observed in the crystal structure agrees well with the results from SEC analysis, as well as analysis of the dimer interface using *PDBePISA* v.1.52 (Krissinel and Henrick, 2007)^6^. PISA calculations returned a CSS (complexation significance score) of 1.0, which confirms a stable homodimeric association. Homodimer formation is associated with a solvation free energy gain (ΔG^int^) of −6.0 kcal mol^−1^ and the free energy of assembly dissociation (ΔG^diss^) of 2.3 kcal mol^−1^. The stability of the dimer is supported by 19 hydrogen bonds and eight salt bridges across the interface, and an interface area of 1366.6 Å^2^. A total of 2,730 Å^2^ of the solvent-accessible surface area of the *Lb*FAE monomers are buried upon dimer assembly. Possible interface salt bridges involve Arg42 Nη1, Nη2, Nε with the Glu73 oxygen atoms Oε1 and Oε2; Arg65 Nη1 and Nη2 with Glu73 Oε2. Electrostatic potential surfaces calculated at the pH optimum of activity (pH 6.5) shows that the buried active site has a net positive charge, whereas the area around the entrance is negatively charged (Figure 5B).

### Binding of ferulic acid

A variant with the catalytic serine replaced by an alanine (S114A) was generated to enable binding of different alkyl hydroxycinnamate compounds. Several S114A crystals were soaked with substrates, including MF and MS, but no binding was observed in the crystal structures. Thus, the refined crystal structure of the MS-soaked S114A variant instead contains an acetate molecule in the active site that originates from the buffer. Wild-type crystals soaked with substrates were also screened, but only FA (i.e., the product) resulted in a complex with *Lb*FAE.

**Figure 6:**
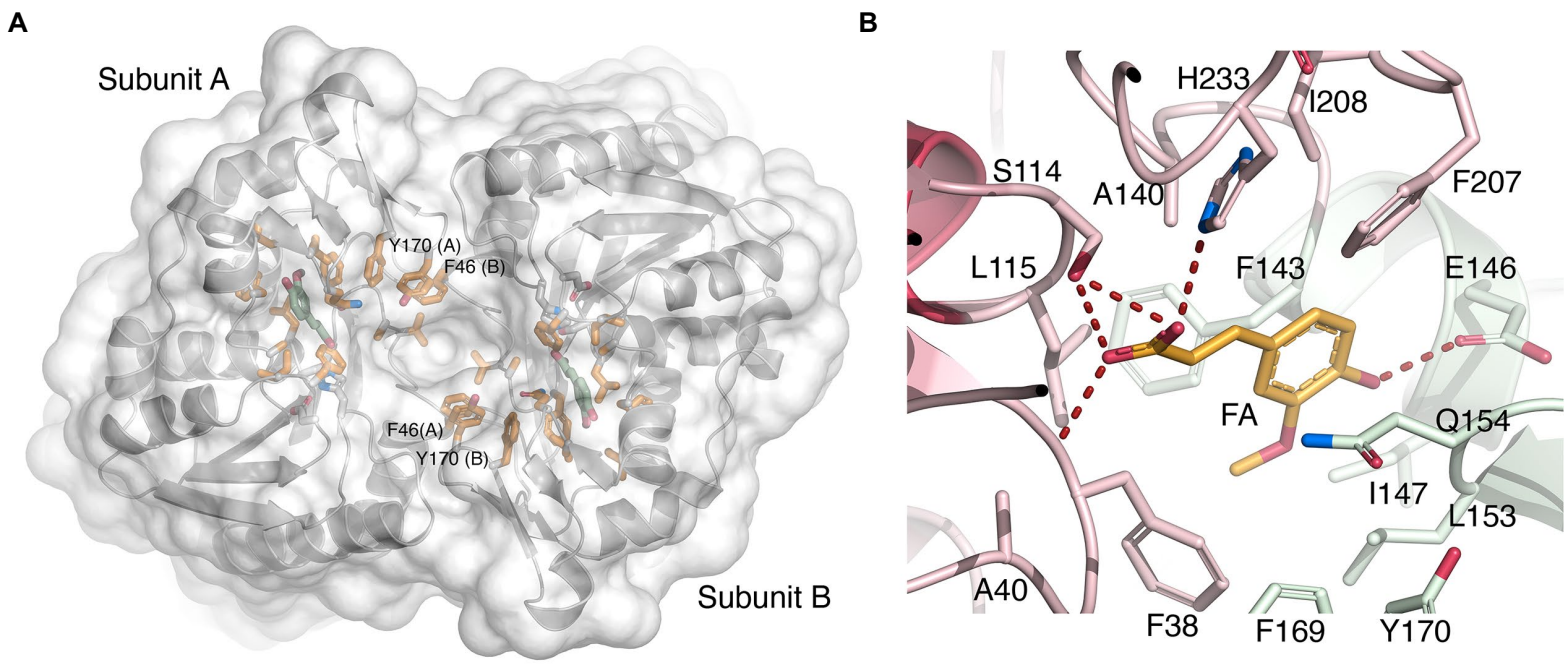
Binding of ferulate to *Lb*FAE. **(A)** Ribbon drawing of the *Lb*FAE homodimer with an overlaid water-accessible surface viewed from the entrance to the active-site groove (view from bottom as in Figure 4B). The ferulate molecule is colored green, and hydrophobic residues lining the groove are colored orange. An arc of aromatic residues from each monomer (Phe207-Phe38-Phe169-Tyr169-Phe46) form an oval collar around the active-site entrance. The narrow opening to the active sites is seen as a deep indentation at the center of the dimer. **(B)** Details of the key enzyme-ligand interactions. The ferulate molecule is colored orange, and residues belonging to the core and lid domains are colored red and green, respectively. See text for additional details.

As mentioned above, the two active sites are only accessible from one face of the homodimer, the other end being closed off by close-packed side-chain atoms. When viewed down the entrance, aromatic side chains are observed to line the entrance opening such that Phe207-Phe38-Phe169-Tyr169-Phe46 from each monomer together form an oval, almost closed collar (Figure 6A). Both carboxylic oxygen atoms O1 and O2 of FA are within hydrogen-bonding distance of Ser114 Oγ (Figure 6B). Moreover, FA O1 can hydrogen bond to the backbone amide group of Phe38, and FA O2 can form an ionic bond with His233 Nε2.

Additionally, the O4 hydroxyl group of the cinnamoyl ring offers a hydrogen bond to Glu146 Oε1 (Figure 6B). Hydrophobic interactions with the cinnamoyl ring of FA involves Phe143 Cα and Cβ, and Gln154 Cγ, and the C10 atom of the methoxy substituent which engages in a CH/π interaction with the nearby Phe38 ring. The unliganded wild-type and S114A structures do not reveal significant conformational changes compared with the FA-bound wild-type model, which suggests that binding of substrate, and release of product, may not require major structural rearrangements.

### Structural comparison with related enzymes

Sequence searches using *BLASTP*^7^ to find similar FAEs with known experimental 3D structure returned the cinnamoyl esterase LJ0536 from *L. johnsonii* as the closest match (Lai et al., 2011), which was therefore used as search model for molecular replacement calculations. The refined *Lb*FAE model in complex with FA was submitted to the *DALI* server (Holm, 2020)^8^ to identify similar 3D structures. The search returned only two models with root-mean-square deviation (r.m.s.d.) values less than 2.0 Å, namely the expected *L. johnsonii* feruloyl esterase LJ0536 (PDB 3PFB, Lai et al., 2011; r.m.s.d. 1.5 Å; 30% sequence identity), and feruloyl esterase Est1E from *Butyrivibrio proteoclasticus* (PDB 2WTN, Goldstone et al., 2010; r.m.s.d. 1.9 Å; 26% sequence identity).

**Figure 7:**
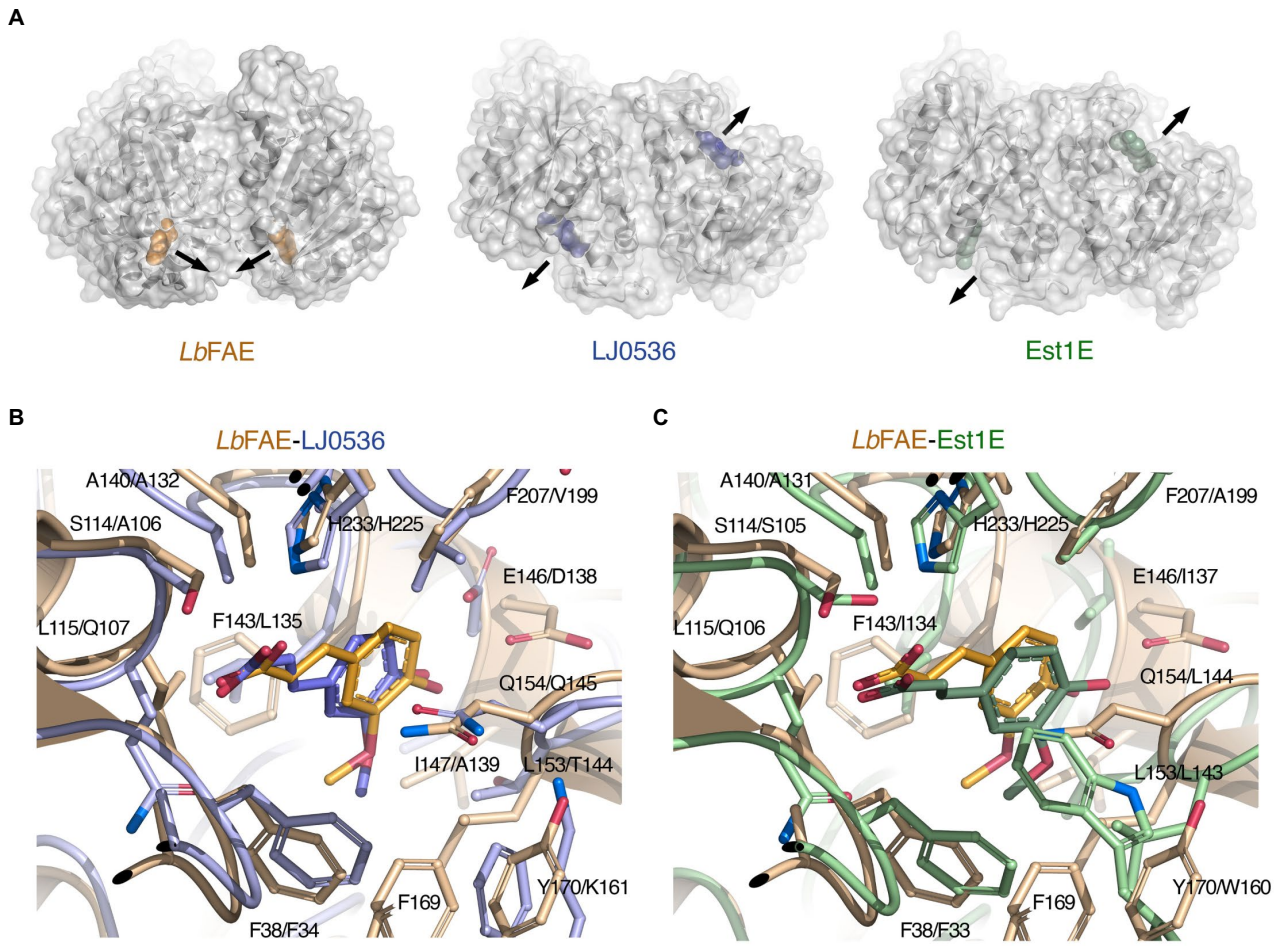
Comparison of *Lb*FAE with the closest known FAE structures. **(A)** Ribbon drawings with overlaid molecular surfaces for *Lb*FAE in complex with ferulate in orange, *L. johnsonii* cinnamoyl esterase LJ0536 in complex with ethylferulate in blue (PDB 3PFB) and *B. proteoclasticus* Est1E in complex with ferulate in green (PDB 2WTN). For the LJ0536 and Est1E dimers, the active sites are situated on opposite faces of the homodimer and are surface-exposed. In *Lb*FAE, the active sites are facing each other and are buried below the protein surface. The arrows indicate the active-site entrances. Overlaid active site in the wild-type *Lb*FAE ferulate complex (orange) with the **(B)** S106A-LJ0536 ferulate complex (blue; PDB 3PFC), and with the **(C)** wild-type Est1E ferulate complex (green; PDB 2WTN). The first residue in each label is for *Lb*FAE. The ferulate molecule assumes identical orientations but slightly different conformation in *Lb*FAE and LJ0536, whereas the ferulate molecule in Est1E has a different orientation in relation to the active site.

LJ0536 and Est1E form homodimers, and interestingly, these homodimers are fundamentally different from that of *Lb*FAE, with their shallow and open active sites exposed toward the solvent rather than facing each other (Figure 7A). Our *PDBePISA* calculations on 2WTN shows that the Est1E dimer in the crystal is predicted to be stable (CSS 1.0), but with a modest ΔG^int^ of −0.2 kcal mol^−1^. The authors report that results from dynamic light scattering were consistent with a dimeric Est1E assembly (Goldstone et al., 2010). According to *PDBePISA*, the LJ0536 dimer (3PFB) is not likely to represent a stable assembly (CSS 0.0), and no experiments were reported that confirm that LJ0536 is a dimer in solution. Several other crystal structures of FAEs have been shown to form stable, homodimeric assemblies (Supplementary Table S2), but these have monomer structures that are more distantly related to *Lb*FAE, LJ0536 and Est1E.

There are 14 unique experimental 3D structures available for bacterial enzymes with predicted FAE activity (one additional structure has not been released), and five unique structures corresponding to fungal FAEs (Supplementary Table S2). To visualize the phylogenetic relationship between these enzymes the amino-acid sequences were aligned based on the best matching of their corresponding atomic 3D coordinates to generate a structure-based sequence alignment with *PDBeFold* (Supplementary Figure S2). This type of alignment provides correct matching of non-identical residues, gaps, and insertions, which is particularly important when the sequence identity is low, as in the case of FAEs. Indeed, the low sequence similarity is evident from the alignment with only five residues strictly conserved across the 19 FAE members (Supplementary Figure S2). The resulting phylogenetic tree confirms LJ0536 and Est1E as the closest neighbors to *Lb*FAE among FAEs with known 3D structure (Supplementary Figure S3).

A structural alignment using only the three closest structures *Lb*FAE (FA complex, chain A, tag removed), LJ0536 (PDB 3PFC, FA complex, chain A, tag removed) and Est1E (PDB 2WTN, FA complex, chain A, tag removed) reveals 38 shared identical residues with an overall r.m.s.d. of 1.42 Å (Supplementary Figure S4). Pairwise structural comparisons give the following r.m.s.d. and sequence identity: *Lb*FAE-LJ0536 of 1.45 Å and 31.2%; *Lb*FAE-Est1E 1.66 Å and 26.9%; and *LJ*0536-Est1E 1.10 Å and 31.2%. If instead chain B of Est1E entry 2WTN is used, r.m.s.d. and sequence-identity values for *Lb*FAE-Est1E are 1.69 Å and 27.0%, and for the *LJ*0536-Est1E pair 1.05 Å and 31.3%.

Although the active sites in *Lb*FAE, LJ0536 and Est1E bear obvious similarities, including the catalytic triad and the G-X-S-X-G motif, there are also differences that indicate differences in substrate specificity (Supplementary Figure S4). As already noted above, the active sites in LJ0536 and Est1E are shallow and surface-exposed (Figure 7A), but in *Lb*FAE is accessible only from a central opening of the homodimer. Beyond the difference in active-site accessibility, the precise conformations of the lid loops that covers the active sites differ, as do the identities and conformation of several key side chains. Of the two homologs, the active site in LJ0536 is clearly more similar to *Lb*FAE than Est1E is. The most pronounced conformational differences that directly affect ligand binding occur in the β7-α4 loop (residues 204–209) where the catalytic histidine is situated, and in the first half (residues 139–169) of the lid domain. Another important difference relates to the different dimerization mode in *Lb*FAE, where Phe46 from subunit B reaches into the active site of subunit A to stack with Tyr170 in subunit A (Figure 6A) and spatially restrict the active site compared with the more exposed active sites in the homologs.

There are more obvious differences at the detailed structural level. In both *Lb*FAE and LJ0536, the FA molecule is bound in the same orientation and conformation in both subunits of the homodimers, whereas in Est1E, the FA is bound in two distinctly different orientations in the active site of subunits A and B, where the binding mode observed for Est1E subunit B agrees with that in LJ0536 and *Lb*FAE. Thus, the FA molecule in *Lb*FAE, LJ0536 (PDB 3PFC; Figure 7B) and Est1E subunit B (PDB 2WTN chain B; Figure 7C) have the same orientation. The only difference is the precise conformation with the C2-C1-C6-C8 dihedral angle being in cis configuration (0°) in *Lb*FAE, and trans configuration (180°) in LJ0536 and Est1E (Figures 7B,C).

While several residues are identical (Figures 6B,C; Supplementary Table S3), the precise side-chain conformation typically differ somewhat, and in Est1E particularly, the different orientation of the FA molecule in chain A engages interactions not observed in *Lb*FAE and LJ0536. Leu144 in Est1E chain B is probably the most obvious example, where the leucine side chain in Est1E moves to assume the position of the cinnamoyl ring in *Lb*FAE, LJ0536 and Est1E chain B.

Despite about 40% of the active-site residues being identical between the three enzymes (Supplementary Table S3), the emerging picture suggests a more hydrophobic active-site cavity in *Lb*FAE, which is mainly due to the amino-acid replacements at positions 115, 143, 147, 153, 178, and 207 (indicated by an asterisk in Supplementary Table S3). As discussed above, the hydrophobic character of the *Lb*FAE active site is further emphasized by an oval collar of Phe residues around the central entrance between the two subunits of the homodimer (Figure 6A).

Structures of complexes with different ligands have been determined for LJ0536, namely FA (PDB 3PFC), ethyl ferulate (PDB 3PFB, 3QM1) and caffeate (PDB 3S2Z; Lai et al., 2011). In the LJ0536-FA complex, Asp138 Oδ2 can form a hydrogen bond to the C4 hydroxyl group of the hydroxycinnamoyl ring (Glu146 Oε1 in *Lb*FAE). This appears to be a suitable candidate for hydroxycinnamate versus cinnamate selectivity. Lai and co-workers confirmed by site-directed mutagenesis that both Asp138 and Gln145 are critical for substrate recognition (Glu146 and Gln154 in *Lb*FAE). As for Gln154 in *Lb*FAE, Gln145 in LJ0536 interacts with its Cγ hydrogen atoms with the hydroxycinnamoyl ring through CH/π interaction. In Est1E, Asp138 in LJ0536 (Glu146 in *Lb*FAE) is occupied by Ile137, which is unable to interact with the hydroxycinnamoyl ring. Moreover, Gln145 in LJ0536 (Gln154 in *Lb*FAE) is replaced by Leu144 in Est1E which can only offer van der Waals interactions with the O3-C10 group of FA. These differences may suggest a different substrate-ring selectivity mechanism in Est1E.

All three enzymes can provide suitably similar, but not identical, hydrophobic interactions with the ethyl moiety of the hydroxycinnamoyl ethoxy group. However, this region in Est1E differs substantially between the two subunits. In subunit A, two lid loops (residues 142–148 and 158–164) rearrange to flip FA toward the entrance of the active site. This type of flexibility is not observed in our structures of unbound and FA-bound *Lb*FAE, and neither in the structures of LJ0536 (Lai et al., 2011). In *Lb*FAE, the lid loop 168–173, which corresponds to the flexible lid loop 158–164 in Est1E, is part of the dimer interface in *Lb*FAE.

This is not the case for Est1E, which forms a different type of dimer. Therefore, we consider a similar conformational change of the lid loop and reorganization of the FA product unlikely in *Lb*FAE since it would disrupt the *Lb*FAE homodimer. A conformational change corresponding to that in Est1E has also been deemed unlikely for LJ0536 (Lai et al., 2011). Taken together, the absence of the hydroxycinnamoyl-selectivity determinants in Est1E present in both *Lb*FAE and LJ0536, and the highly flexible and surface-exposed lid loops further suggest that the substrate specificity and selectivity of Est1E is fundamentally different.

## Discussion

In this work, we have shown that the structure of *Lb*FAE reveals a new type of dimerization compared with previously characterized bacterial homodimeric FAEs. Correlating the structural details to differences in substrate specificity and enzyme function is challenging since the substrates used for biochemical characterization of the three enzymes *Lb*FAE, LJ0536 and Est1E differ, and all enzymes have slightly different structural determinants for substrate binding. Est1E is produced by the rumen bacterium *B. proteoclatsicus* and displays broad activity on plant-derived substrates, including ethyl ferulate, ethyl cinnamate, ethyl-3-coumarin carboxylate, ryegrass hemicellulose and birchwood xylan with the highest specific activity observed for ethyl-3-coumarin carboxylate (Goldstone et al., 2010).

*In-silico* docking of arabinofuranose to Est1E suggested that an arabinofuranosyl group attached to a hydroxycinnamoyl compound can be accommodated in the active site, and that the apparent flexibility of loops in the lid domain would allow large substrates. In the case of *Lb*FAE however, the restricted access to the active sites and seemingly low flexibility in the lid domain suggest that the natural substrate is limited to accessible arabinofuranosyl-ferulate side chains on arabinoxylan chains. This agrees with our observations that *Lb*FAE did not produce diferulic acid, and that relatively low amounts of FA were released from F-AX (Figure 3). The limited accessibility of the active site of *Lb*FAE would probably make this enzyme inefficient on lignin-ferulate-polysaccharide complexes.

**Figure 8:**
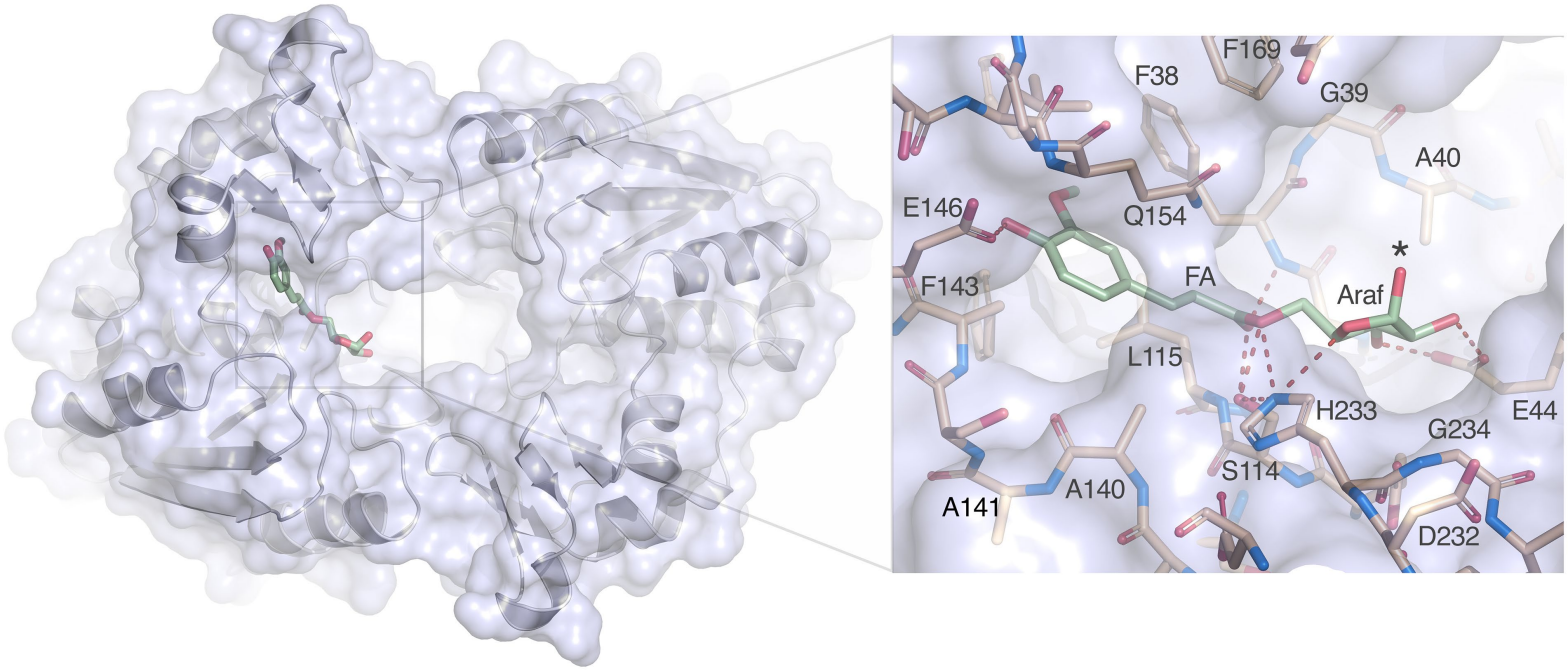
Docking of arabinofuranosyl ferulate. Arabinofuranosyl ferulate (Araf-FA; green) docked in the active site of *Lb*FAE. The view is from the bottom of the homodimer where the entrance to the active site is located. The arabinofuranosyl moiety is exposed to the opening at the dimer interface. The inset shows possible hydrogen bonds between the ferulate and arabinofuranosyl moieties as dashed red lines.

Nevertheless, docking of arabinofuranosyl ferulate (Araf-FA) to the *Lb*FAE active site is straightforward. Despite the restricted active site and narrow opening between the two subunits, the Araf moiety is easily accommodated and is positioned in open cavity leading to the active sites (Figure 8). Several favorable interactions are possible with Glu146 coordinating the hydroxycinnamoyl hydroxyl group, Ser114 and His233 can provide hydrogen bonds to the ester oxygen of the bond to be cleaved, and Glu44 can form two hydrogen bonds to the O2’ and O3 hydroxyl groups of the arabinofuranosyl moiety of the leaving group. In addition, His233 is within hydrogen-bonding distance to the nucleophilic Ser114 and to the endocyclic O4’ oxygen of the arabinofuranosyl ring. Thus, the substrate is anchored in three critical positions, the aromatic ring, the ester oxygen and the sugar ring.

While arabinofuranosyl ferulate is well accommodates, how *Lb*FAE accesses this substrate also needs to be considered. We therefore modeled a xylotetraose with arabinofuranosyl ferulate linked to the reducing-end xylose residue (Supplementary Figure S5). The architecture of the homodimer makes it likely that *Lb*FAE approaches xylan chain ends decorated with arabinofuranosyl ferulate with an exo-type approach, rather than cleaving arabinofuranosyl ferulate groups attached internally in the xylan backbone. In this binding mode, the hydroxyl group of Tyr170 could form a hydrogen bond with O3 of the second xylose residue, and the third xylose residue would stack favorably with the ring of Phe207 (Supplementary Figure S5). It is possible that conformational changes in *Lb*FAE upon encountering the polymeric substrate would allow also internal arabinofuranosyl ferulate groups to be cleaved.

A histidine-ring flip mechanism has been proposed based on NMR studies for the protonation of the leaving group (Ash et al., 2000). Recently, results from quantum mechanics/molecular mechanics (QM/MM)-based transition-path sampling (TPS) on *A. niger* FaeA has provided strong support for an alternative mechanism whereby the histidine shuttles a proton from the nucleophile to the leaving group as a concerted event without ring flip (Silveira et al., 2021). In our proposed interaction network, His233 would indeed be suitably oriented to shuttle a proton from Ser114 to the leaving group in a concerted mechanism without the need for conformational changes.

## Data availability statement

The data presented in the study are deposited in the Protein Data Bank repository (http://www.rcsb.org), accession numbers 7Z2X, 7Z2U, and 7Z2V.

## Author contributions

CD and KK conceived the research. KK, DK, TR, AJ-Q, FV, and CD designed and performed experiments and analyzed the data. All authors contributed to the article and approved the submitted version.

## Funding

This study was partially supported by the Swedish Research Council Formas (Grant number 2016–01449) to KK and additionally enabled by faculty funding to CD. FV acknowledges the Swedish Research Council Formas (Grant number 942–2016-119) for financial support.

## Conflict of interest

The authors declare that the research was conducted in the absence of any commercial or financial relationships that could be construed as a potential conflict of interest.

## Publisher’s note

All claims expressed in this article are solely those of the authors and do not necessarily represent those of their affiliated organizations, or those of the publisher, the editors and the reviewers. Any product that may be evaluated in this article, or claim that may be made by its manufacturer, is not guaranteed or endorsed by the publisher.

## Footnotes

^1^ https://server.PoissonBolzmann.org

^2^ https://www.ebi.ac.uk/msd-srv/ssm/

^3^ https://espript.ibcp.fr/ESPript/ESPript/

^4^ https://www.ebi.ac.uk/Tools/phylogeny/simple_phylogeny

^5^ https://itol.embl.de

^6^ https://www.ebi.ac.uk/pdbe/pisa

^7^ https://blast.ncbi.nlm.nih.gov/Blast.cgi

^8^ http://ekhidna2.biocenter.helsinki.fi/dali/
