# Supplementary material for: Crystal structure of the feruloyl esterase from Lentilactobacillus buchneri reveals a novel homodimeric state
Source: Front Microbiol. 2022 Dec 8;13:1050160. doi: 10.3389/fmicb.2022.1050160 (PMC9776664; doi:10.3389/fmicb.2022.1050160)
Supplement: Supplementary file 1 [file Data_Sheet_1.PDF]

Supplementary material

Figure S1

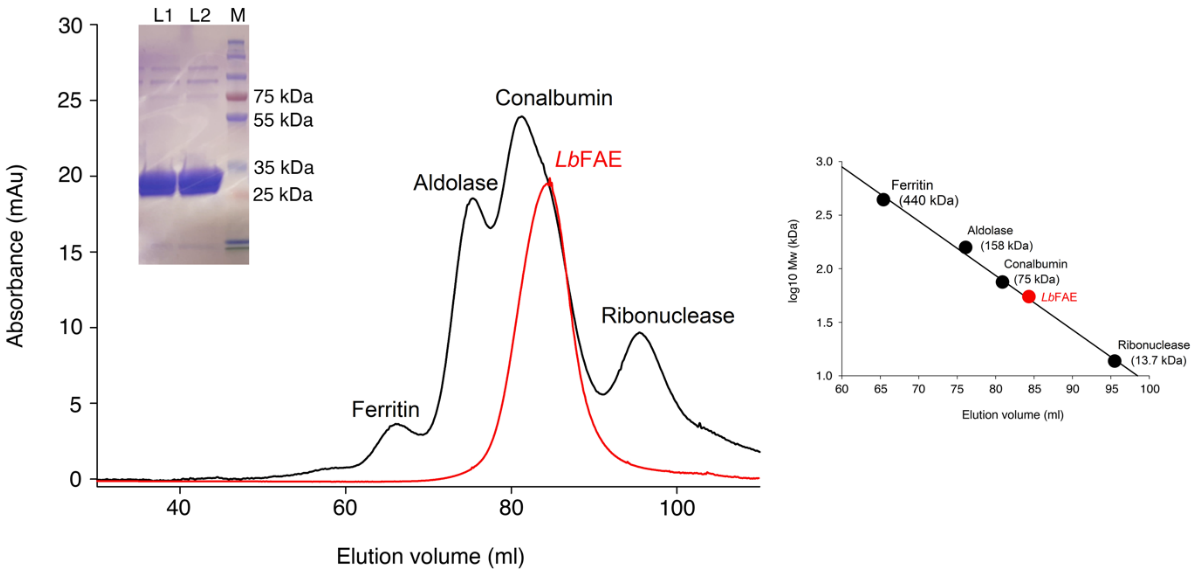

FIGURE S1

SDS-PAGE and SEC analysis. Monomer molecular weight determined by SDS PAGE, and SEC profile consistent with a homodimer.

Figure S2

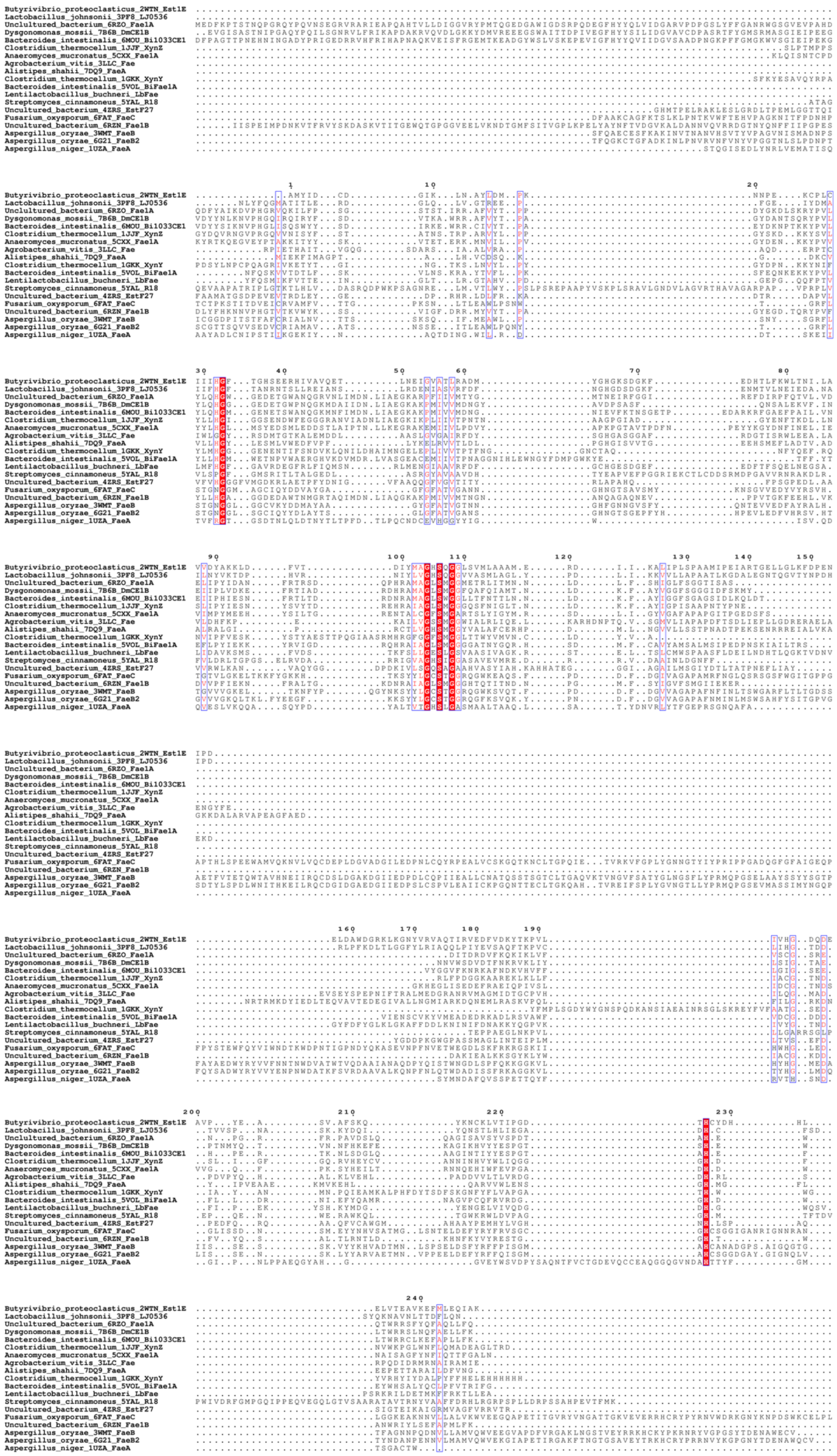
Butyrivibrio_proteoclasticus_2WTN_Est1E
Lactobacillus_johnsonii_3PF8_LJ0536
Uncultured_bacterium_6RZO_FaelA
Dysgonomonas_mossii_7B6B_DmCE1B
Bacteroides_intestinalis_6MOU_Bi1033CE1
Clostridium_thermocellum_1JJF_XynZ
Anaeromyces_mucronatus_5CXX_FaelA
Agrobacterium_vitis_3LLC_Fae
Alistipes_shahii_7DQ9_FaeA
Clostridium_thermocellum_1GKK_XynY
Bacteroides_intestinalis_5VOL_BiFaelA
Lentilactobacillus_buchneri_LbFae
Streptomyces_cinnamoneus_5YAL_R18
Uncultured_bacterium_4ZRS_EstF27
Fusarium_oxysporum_6FAT_FaeC
Uncultured_bacterium_6RZN_FaelB
Aspergillus_oryzae_3WMT_FaeB
Aspergillus_oryzae_6G21_FaeB2
Aspergillus_niger_1UZA_FaeA

FIGURE S2

Structure-based sequence alignment for FAEs with known 3D structure. 3D-aligned models used to generate the sequence alignment included 14 bacterial structures: *Lb*FAE-ferulate complex (this work), 3PF8, 2WTN, 3LLC, 4ZRS, 5YAL, 7B6B, 6MOU, 5RXO, 6RZN, 1JJF, 5VOL, 1GKK and 7DQ9; and 5 fungal structures: 3WMT, 6G21, 6FAT, 1UZA and 5CXX. Chain A of each model was used.

Figure S3

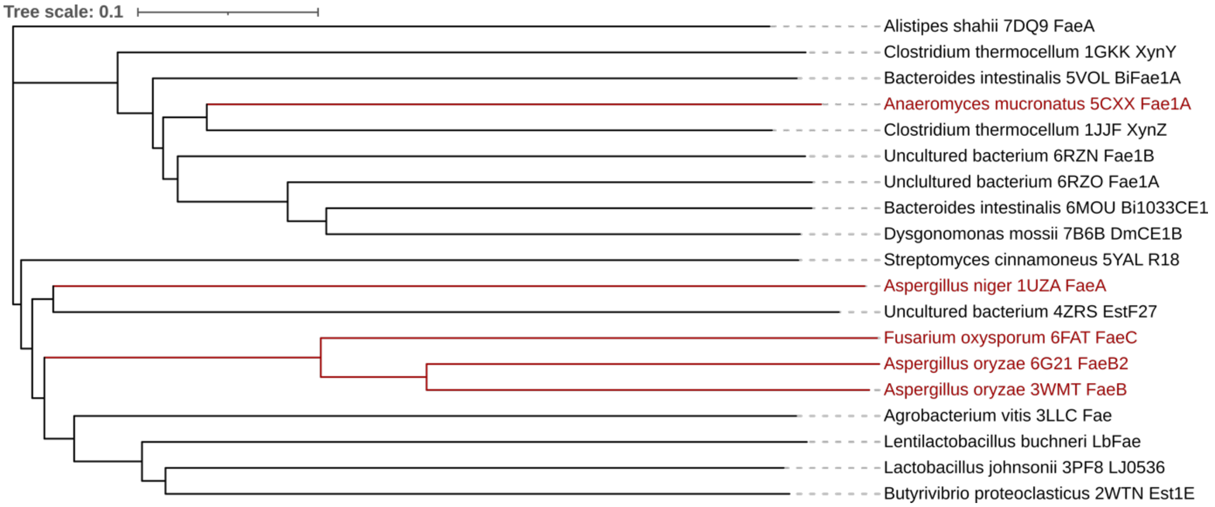

FIGURE S3

Phylogenetic tree of FAEs with known 3D structure. Phylogenetic tree calculated from a structure-based MSA. Bacterial and fungal members are colored black and red, respectively. The PDB accession codes used for structure-based sequence alignment are indicated in the picture.

Figure S4

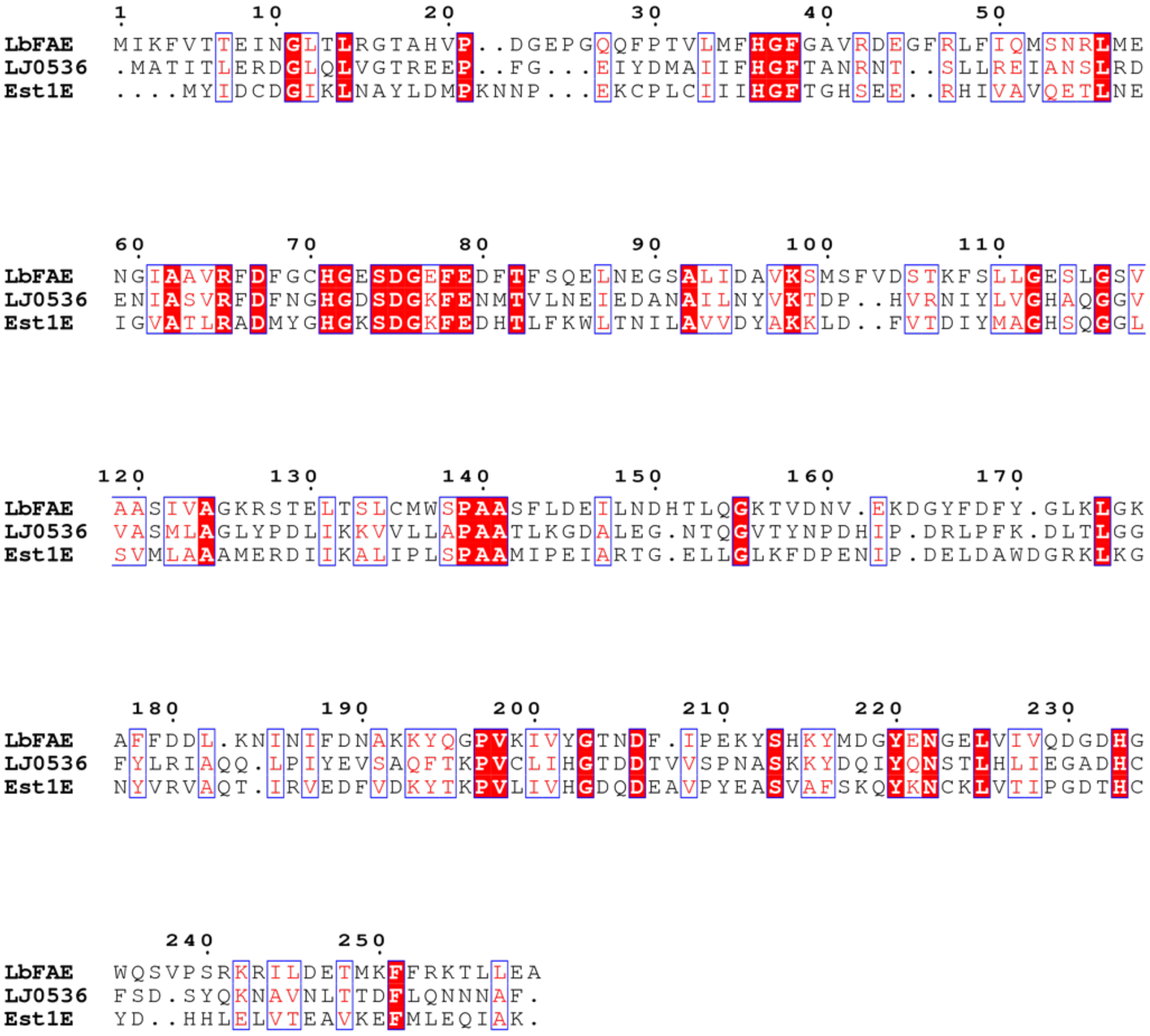

FIGURE S4

Structure-based sequence alignment for *Lb*FAE, LJ_0536 and Est1E. Sequence alignment based on structural alignment of *Lb*FAE, *L. johnsonii* LJ_0536 (PDB 3PF8, chain A) and *B. proteoclasticus* Est1E (PDB 2WTN, chain A).

Figure S5

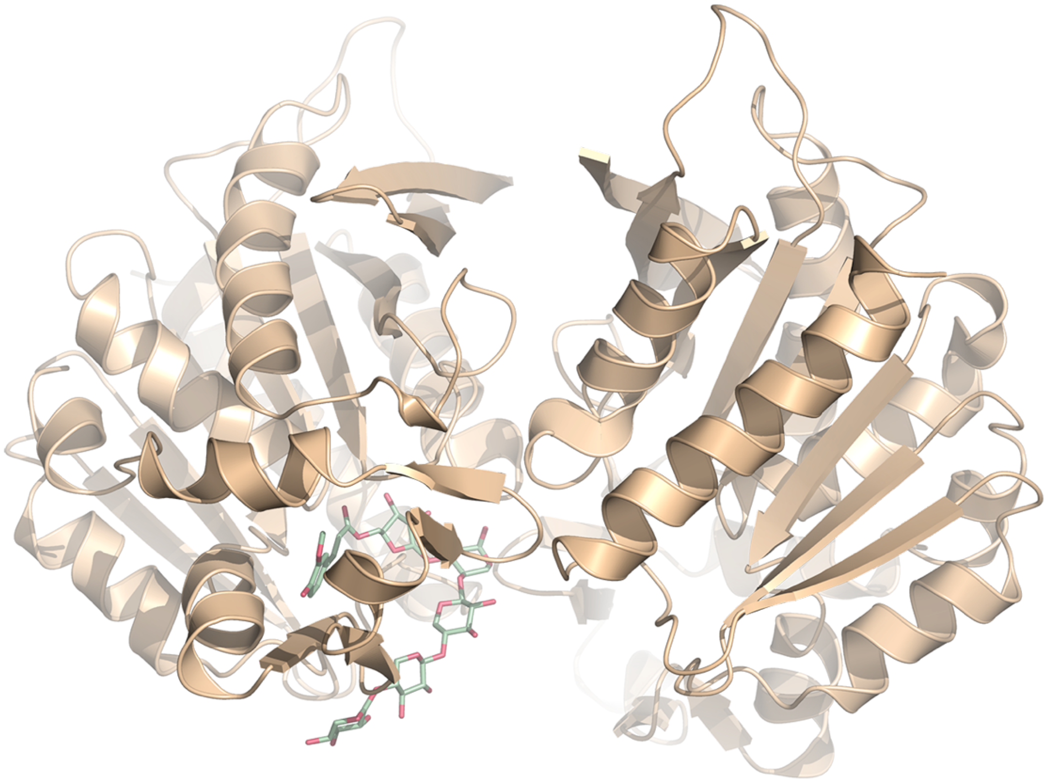

FIGURE S5

Ribbon diagram showing the *Lb*FAE homodimer. A xylotetrasaccharide has been modeled with a arabinofuranosyl ferulate side chain at its reducing end that is reaching into the active site of one subunit.

Table S1. Data collection, phasing and refinement statistics

**Data collection**[a]	Wild type	Wild type, ferulate complex	S114A
Synchrotron, beamline	Diamond i24	Bessy BL14-2	Diamond I03
Wavelength (Å)	0.96852	0.91840	0.97629
Space group / mol per a.s.u.	*P*1 (1) / 4	$P4_32_12$ (96) / 2	$P4_32_12$ (96) / 2
Cell dimensions: *a*, *b*, *c* (Å), α, β, γ (°)	47.83, 62.23, 94.85, 103.054, 101.523, 92.801	79.50, 79.50, 228.24, 90, 90, 90	79.96, 79.96, 227.94, 90, 90, 90
Resolution (Å), nominal	30.93–1.50 (1.60–1.50)	46.36–1.90 (2.00–1.90)	46.41–1.45 (1.50–1.45)
Unique reflections	155,659 (26,396)	58,652 (5738)	130,627 (11,811)
R_{sym}	0.111 (0.668)	0.256 (2.482)	0.069 (1.754)
$I / \sigma I$	6.7 (2.0)	13.4 (1.5)	25.1 (1.2)
Completeness (%)	93.5 (90.0)	99.9 (99.8)	99.3 (94.5)
Redundancy	2.8 (2.3)	25.1 (22.9)	22.5 (9.0)
CC(1/2)[b]	98.8 (52.8)	99.8 (61.3)	100.0 (52.6)
Wilson *B* factor (Å^2)	14.41	25.21	19.76
**Refinement**			
Resolution (Å)	30.16–1.50 (1.55–1.50)	46.36–1.90 (1.97–1.90)	40.14–1.45 (1.50–1.45)
Completeness	93.5 (89.9)	99.86 (99.72)	99.33 (94-67)
No. reflections work	155,651 (14,967)	58,639 (5738)	130,607 (12,237)
No. reflections free	1985 (195)	2000 (196)	2000 (187)
R_{work}	0.182 (0.260)	0.180 (0.308)	0.167 (0.328)
R_{free}	0.211 (0.285)	0.217 (0.354)	0.177 (0.334)
N°. non-hydrogen atoms: all, protein, water, ligands	9263, 8384, 879, –	4554, 4163, 333, 58	4802, 4210, 575, 17
N°. protein residues, all chains	1050	526	525
Mean *B* factors protein, water, ligands (Å^2)	16.2, 24.8, –	25.7, 30.9, 31.0	22.9, 33.0, 28.4
R.m.s deviation bond lengths (Å), bond angles (°)	0.005, 0.81	0.010, 0.93	0.005, 0.77
Ramachandran, MolProbity: allowed, favored, outliers (%)	99.71, 97.50, 0.22	100, 97.13, 0.00	99.81, 97.12, 0.19
PDB ID	7Z2X	7Z2U	7Z2V

[a] Statistics for outer-shell reflections are given in parentheses.

[b] Percentage of correlation between intensities from random half-datasets as given by XSCALE. Values given represent correlations significant at the 0.1% level [Karplus & Didrichs 2012].

Table S2. Predicted feruloyl esterases with known three-dimensional structure

Bacteria	PDB accession	UNP accession	Signal peptide	Variant	Oligomer	Dimer stability, CSS	Functional ligand	Catalytic triad	G-X-S-X-G motif	Km (mM), kcat (1/s), Vmax	Comments	Reference
Streptomyces cinnamoneus NBRC 12852, R18	5YAE	A0A0M4UW33	Tat/SPI 0.64	WT	Monomer	–	–	Ser191, His268, Asp214	(189) G-H-S-I-G (193)	MF: 5.59, 2.53, –; MS: 2.56, 0.10, –; EF: 3.72, 1.70, –		https://doi.org/10.1128/AEM.02300-17
	5YAL	A0A0M4UW33		WT	Monomer	–	Ethyl ferulate (not bound)					https://doi.org/10.1128/AEM.02300-17
Clostridium thermocellum, endo-1,4-b-xylanase Y	1GKK	P51584	Sec/SPI 0.99	WT	Monomer	–	–	Ser954, His1028, ASP1018	(952) G-F-S-M-G (956)	–	Cannot accommodate ferulate crossbridge	https://doi.org/10.1016/s0969-2126(01)00684-0
	1GKL	P51584		S954A	Monomer	–	Ferulate			–	No lid, instead packing against GH domain	https://doi.org/10.1016/s0969-2126(01)00684-0
	1WB4	P51584		S954A	Monomer	–	Sinapate			–		https://doi.org/10.1107/S0907444904029695
	1WB5	P51584		S954A	Monomer	–	Syringate			–		https://doi.org/10.1107/S0907444904029695
	1WB6	P51584		S954A	Monomer	–	Vanillate			–		https://doi.org/10.1107/S0907444904029695
Clostridium thermocellum, endo-1,4-b-xylanase Z	1JJF	P10478	Sec/SPI 0.97	WT	Monomer	–	–	Ser172, His260, Asp230	(170) G-L-S-M-G (174)	–	Open and solvent exposed ferulic acid binding site suggests	https://doi.org10.1021/bi011391c
	1JT2	P10478		S172A	Monomer	–	Ferulate			–	No lid, binds slightly different comp to LbFAE	https://doi.org10.1021/bi011391c
Bacteroides intestinalis DSM 17393, Bi1033-CE1	6MOT	B3C969	Sec/SPI 0.95	WT	Monomer	–	–	Ser273, His365, Glu336	(271) G-L-S-W-G (275)	Low ferulic acid cleavage activity on feruloylated monosaccharide (	Esterase-gene-enriched PUL (EGE PUL)	https://doi.org/10.1038/s41467-020-20737-5
	6MOU	B3C969		WT	Dimer	Yes, 1.00	–			–	No lid	https://doi.org/10.1038/s41467-020-20737-5
Alistipes shahii WAL 8301	7DQ9	D4INH0	None	WT	Monomer	–	–	Ser96, His245, Asp217	(94) G-H-S-M-G (98)	–	Lid 126-195, Type-A FAE	n.a.
Dysgonomonas mossii DSM 22836, DmCE1B	7B5V	F8X1N1	Sec/SPI 0.68	WT	Dimer	Yes, 1.00	–	Ser542, His638, Glu606	(549) G-L-S-M-G (544)	MS: 0.36, 5.0, –; pNP-Ac: 3.5, 12.9, –; MF and MC not saturable up	Present in PUL, CE1 joined to CBM48	https://doi.org/10.1016/j.jbc.2021.100500
	7B6B	F8X1N1		WT	Dimer	Yes, 1.00	Ferulate				No lid, dimer mediated by b1 and CBM	https://doi.org/10.1016/j.jbc.2021.100500
Uncultured bacterium, CE1-GH62-GH10, Fae1A	6RZO	–	Unknown	WT	Dimer	Yes, 1.00	–	Ser242, His325, Glu296	(270) G-L-S-M-G (274)		No lid, dimer mediated by b1 and cbm	https://doi.org/10.1074/jbc.RA119.009523
Uncultured bacterium, CE1-GH62-GH10, Fae1B	6RZN	–	Unknown	WT	Dimer	Yes, 1.00	–	Ser272, His368, Asp339	(240) G-L-S-M-G (244)		No lid, dimer mediated by b1 and CBM	https://doi.org/10.1074/jbc.RA119.009523
Uncultured bacterium, EstF27	4ZRS	E7DJY5	Unknown		Dimer	Yes, 1.00	–	Ser151, His263, Asp230	(149) G-Q-S-A-G (153)		No lid, Back-to-back dimer mediated by b8	https://doi.org/10.1021/acs.jafc.5b03424
Lactobacillus buchneri, feruloyl esterase LbFAE	To be deposited	D7RU28	None	WT	Dimer	Yes, 1.00	–	Ser114, His233, Asp206	(112) G-E-S-L-G (116)	?	Lid 143-186	This work
Lactobacillus johnsonii, cinnamoyl esterase LJ0536	3PF8	D3YEX6	None	WT	Dimer		–	Ser106, His225, Asp197	(104) G-H-S-Q-G (108)	pNP-but: 0.16, 1.53, 3.34	Different dimer than LbFAE, similar to Est1E	https://doi.org/10.1371/journal.pone.0023269
	3PF9	D3YEX6		S106A	Dimer		–			pNP-but: 0, 0, 0	Back-to-back dimer mediated by a2 and a3	https://doi.org/10.1371/journal.pone.0023269
	3S2Z	D3YEX6		S106A	Dimer		Caffeate					https://doi.org/10.1371/journal.pone.0023269
	3PFB	D3YEX6		S106A	Dimer	No	Ethyl ferulate				less space for ethoxy group in LbFAE	https://doi.org/10.1371/journal.pone.0023269
	3QM1	D3YEX6		S106A	Dimer		Ethyl ferulate					https://doi.org/10.1371/journal.pone.0023269
	3PFC	D3YEX6		S106A	Dimer		Ferulate				Lid 135-176, FA binds as in 1JT2	https://doi.org/10.1371/journal.pone.0023269
Butyrivibrio proteoclasticus, Est1E	2WTM	D2YW37	None	WT	Dimer	Yes, 1.00	–	Ser105, His225, Asp197	(103) G-H-S-Q-G (107)	pNP-Ac: 0.19, 23.0, –; pNP-But 0.24, 24.0, –	Promiscuous, lid domain with new fold	https://doi.org/10.1002/prot.22662
	2WTN	D2YW37		WT	Dimer	Yes, 1.00	Ferulate				Lid 135-176	https://doi.org/10.1002/prot.22662
Agrobacterium vitis S4	3LLC	B9JYM4	None	WT	Monomer	–	–	Ser113, His246, Asp216	(111) G-S-S-M-G (115)	–	Lid 148-195, PEG binds in active site	n.a.
Lactobacillus plantarum ATCC BAA-793, LP_0796	7EBO, not released	F9UM18	None	WT	Monomer	–		Ser94, His225, Asp195		MC: 0.065, 0.089, –		https://doi.org/10.1016/j.ijbiomac.2021.06.033
Bacteroides intestinalis DSM 17393, BiFae1A	5VOL	B3CET1	Sec/SPI 0.87	WT	Tetramer	–	–	Ser152, His265, Asp236	(150) G-L-S-M-G (154)	MF: 0.052, 16.8, –	β-clamp motif, shorter lid	https://doi.org/10.1016/j.jmb.2017.06.017
**Fungi**	**PDB accession**	**UNP accession**	**UNP accession**	**Variant**	**Oligomer**		**Functional ligand**	**Catalytic triad**	**G-X-S-X-G motif**	**Km (mM), kcat (1/s), Vmax**	**Comments**	**Reference**
Anaeromyces mucronatus AmCE1/Fae1A	5CXU	F2YCB6	None	WT	Monomer	–	–	Ser156, His253, Asp222	(154) G-F-S-M-G (158)		No lid, instead long loops, FA binds as in 1TJ2	https://doi.org/10.1042/BJ20151153
	5CXX	F2YCB6		WT	Monomer	–	Ferulate					https://doi.org/10.1042/BJ20151153
Fusarium oxysporum, FaeC	6FAT	A0A1D3S5H0	None	WT	Dimer	Not analyzed	–	Ser201, His452, Asp412	(199) G-C-S-T-G (203)		Larger lid domain with different structure 230-387	https://doi.org/10.1002/1873-3468.13776
Aspergillus oryzae, FaeB	3WMT	Q2UP89	Sec/SPI 0.98	WT	Dimer	Not analyzed	–	Ser203, His457, Asp417	(201) G-C-S-T-G (205)		Larger lid domain with different structure 232-391	https://doi.org/10.1002/prot.24649
Aspergillus oryzae, FaeB2	6G21	Q2UMX6	Sec/SPI 0.92	WT	Dimer	Not analyzed	Ferulate (not bound)	Ser169, His421, Asp381	(167) G-C-S-T-G (171)		Larger lid domain with different structure 198-355	n.a.
Aspergillus niger	1USW	O42807	Sec/SPI 0.99	WT	Monomer	–	–	Ser133, His247, Asp194	(131) G-H-S-L-G (135)		No lid, instead long loops	https://doi.org/10.1016/j.jmb.2004.03.003
	1UWC	O42807		WT	Monomer	–	Ferulate					https://doi.org/10.1107/S0907444904004937
	1UZA	O42807		WT	Monomer	–	–					https://doi.org/10.1107/S0907444904004937
	2BJH	O42807		S133A	Monomer	–	Ferulate					https://doi.org/10.1111/j.1742-4658.2005.04849.x
	2HL6	O42807		WT	Monomer	–	CAPS					https://doi.org/10.1016/j.febslet.2006.09.039
	2IX9	O42807		WT	Monomer	–	CAPS					https://doi.org/10.1016/j.febslet.2006.09.039

Table S3. Active-site residues in *Lb*FAE, LJ0536 and Est1E

***Lb*FAE**	**LJ0536**	**Est1E**
Gly37	Gly33	Gly32
Phe38	Phe34	Phe33
Gly39	Thr35	Thr34
Glu113	His105	His104
Ser114 (catalytic)	Ser106	Ser105
Leu115*	Gln107	Gln106
Ala140	Ala132	Ala131
Ser142	Thr134	Met133#
Phe143*	Leu135	Ile134
Glu146	Asp138	Ile137#
Ile147*	Ala139	Ala138
Leu153*	Thr144	Leu143#
Gln154	Gln145	Leu144#
Phe169	Phe160	Ala159#
Tyr170	Lys161	Trp160#
Phe178*	Tyr169	Tyr169
Phe207*	Thr198	Glu198
Ile208	Val200	Val200
His233 (catalytic)	His225	His225

Identities are gray-shaded. The asterisks indicate side chains that provide a more hydrophobic environment in *Lb*FAE. Residues in Est1E that undergoes large conformational changes in response to substrate orientation are indictaed by a hashtag.
